# Urinary Elimination of Ecdysterone and Its Metabolites Following a Single-Dose Administration in Humans

**DOI:** 10.3390/metabo11060366

**Published:** 2021-06-09

**Authors:** Gabriella Ambrosio, Tasha Yuliandra, Bernhard Wuest, Monica Mazzarino, Xavier de la Torre, Francesco Botrè, Patrick Diel, Eduard Isenmann, Maria Kristina Parr

**Affiliations:** 1 Institute of Pharmacy, Pharmaceutical and Medicinal Chemistry (Pharmaceutical Analysis), Freie Universitaet Berlin, 14195 Berlin, Germany; gabriella@zedat-fu-berlin.de (G.A.); tasha.y@fu-berlin.de (T.Y.); 2 Agilent Technologies, 76337 Waldbronn, Germany; bernhard_wuest@agilent.com; 3 Laboratorio Antidoping FMSI, 00197 Rome, Italy; monica.mazzarino@gmail.com (M.M.); xavier.delatorre@fmsi.it (X.d.l.T.); francesco.botre@unil.ch (F.B.); 4 REDs—Research and Expertise in Anti-Doping Sciences, ISSUL—Institute of Sport Sciences, University of Lausanne, 1015 Lausanne, Switzerland; 5 Department for Molecular and Cellular Sports Medicine, Institute for Cardiovascular Research and Sports Medicine, German Sport University Cologne, 50933 Cologne, Germany; diel@dshs-koeln.de (P.D.); e.isenmann@dshs-koeln.de (E.I.)

**Keywords:** ecdysterone, metabolites, excretion profile, urinary pharmacokinetics

## Abstract

Ecdysterone is a phytosteroid widely discussed for its various pharmacological, growth-promoting, and anabolic effects, mediated by the activation of estrogen receptor beta (ERbeta). Performance-enhancement in sports was demonstrated recently, and ecdysterone was consequently included in the Monitoring Program, to detect potential patterns of misuse in sport. Only few studies on the pharmacokinetics of ecdysterone in humans have been reported so far. In this study, post-administration urine samples in twelve volunteers (single dose of 50 mg of ecdysterone) were analyzed using dilute-and-inject liquid-chromatography–tandem mass spectrometry. Identification and quantitation of ecdysterone and of two metabolites, 14-deoxy-ecdysterone and 14-deoxy-poststerone, was achieved. Ecdysterone was the most abundant analyte present in post-administration urine samples, detected for more than 2 days, with a maximum concentration (C_max_) in the 2.8–8.5 h urine (C_max_ = 4.4–30.0 µg/mL). The metabolites 14-deoxy-ecdysterone and 14-deoxy-poststerone were detected later, reaching the maximum concentrations at 8.5–39.5 h (C_max_ = 0.1–6.0 µg/mL) and 23.3–41.3 h (C_max_ = 0.1–1.5 µg/mL), respectively. Sex-specific differences were not observed. Cumulative urinary excretion yielded average values of 18%, 2.3%, and 1.5% for ecdysterone, 14-deoxy-ecdysterone, and 14-deoxy-poststerone, respectively. Ecdysterone and 14-deoxy-ecdysterone were excreted following first-order kinetics with half-lives calculated with three hours, while pharmacokinetics of 14-deoxy-poststerone needs further evaluation.

## 1. Introduction

**Figure 1:**
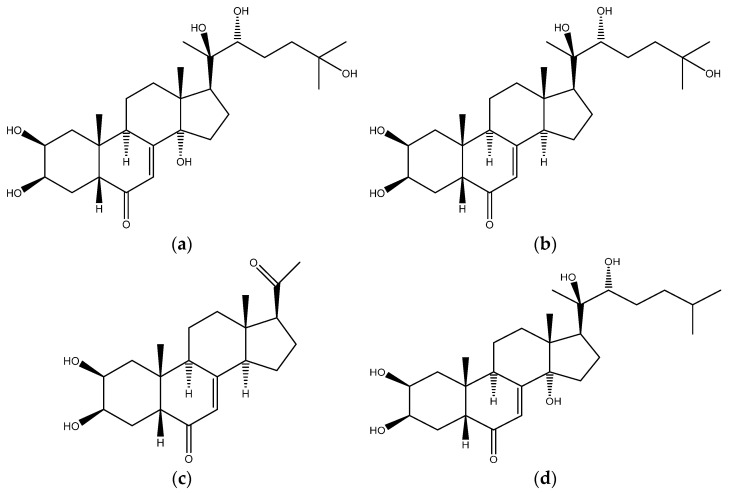
Chemical structure of (**a**) ecdysterone, of (**b**) 14-deoxy-ecdysterone, (**c**) 14-deoxy-poststerone, and (**d**) ponasterone (ISTD).

Ecdysterone (chemical structure Figure 1a) is a steroid hormone naturally present in plants. It is the most widely used active ecdysteroid, and its pharmacological effects have been discussed since the 1980s [1,2]. Studies reported the ability of this natural steroid hormone to stimulate protein synthesis, and change carbohydrate and lipid metabolism [3,4]. It has also been highlighted that ecdysterone is correlated with an increased cell immunity, and that it is also endowed with adaptogenic, anti-diabetic, hepatoprotective, and anti-tumor properties [4,5]. Moreover, growth-promoting and anabolic effects in animals and in humans have been reported [5,6,7,8,9,10,11,12,13,14,15,16,17,18,19,20,21,22,23]. In vitro and in silico studies have shown that the anabolic effect of ecdysterone is mediated by activation of estrogen receptor beta (ER beta) [8,24,25,26,27]. Ecdysterone is marketed as able to increase strength and muscle mass and improve performance, without showing any classical side effects of anabolic androgenic steroids (AAS) [4,5]. For these reasons, the use of dietary supplements containing this “natural” steroid may be considered to be very attractive for athletes aiming to maximize their performance, and it has become a topic of high interest within, but not limited to, the sport context.

Only recently (since 2020), ecdysterone has been included in the Monitoring Program of the World Anti-Doping Agency (WADA), under the section “Anabolic Agents, In-and Out-of-Competition” [28], to assess potential patterns of misuse in sport. This decision has been based mainly on the results of a controlled administration trial in humans, which demonstrated its performance-enhancing effects in power training [29].

Studies on the metabolism of ecdysterone have been conducted mostly in animals and only a few refer to humans [10,30,31,32,33,34]. While ecdysterone does not seem to undergo phase II metabolism [30,34], studies concur on its biotransformation, leading to the formation of dehydroxylated metabolites. However, structure assignment and information on the excretion profiles differ in some studies. Indeed, while Tsitsimpikou et al. reported a 2-deoxy-ecdysterone and deoxy-ecdysone as urinary metabolites [32], Brandt reported the 14-deoxy-ecdysterone [33]. In both studies, the metabolism was evaluated after an administration to a male volunteer of a dietary supplement called “Ecdysten”, for a final ecdysterone content of 20 mg. Blood levels of ecdysterone following the administration of a nutritional supplement containing ecdysterone have also been reported [35].

Recently, in a single dose administration study, 51.5 mg of pure ecdysterone has been administered to one healthy volunteer, and the presence of 14-deoxy-ecdysterone was confirmed in the post-administration urine samples, in comparison to the in-house synthesized reference [34]. Parr et al. reported ecdysterone as the most abundant analyte in post-administration urine samples, with a wider detection window than 14-deoxyecdysterone, which was excreted later [34]. These results are similar to the one reported by Brandt [33], while Tsitsimpikou et al. reported that ecdysterone is detected in urine mainly as deoxy-ecdysone metabolite, followed by the parent compound and the 2-deoxy-ecdysterone metabolite [32].

This study aimed to investigate and provide consistent analytical information regarding the urinary excretion and the pharmacokinetics of ecdysterone and its metabolites in humans. More specifically, we have followed the elimination of ecdysterone and its metabolites, after the administration of a single oral dose of 50 mg in twelve volunteers.

## 2. Results

The analysis of the urine samples has been performed using LC–MS/MS and the mass spectrometer was operated in multiple reaction monitoring (MRM) acquisition mode, using positive ionization (ESI+). The protonated molecular ion [M+H]^+^ for ecdysterone was detected at *m*/*z* 481.3, for 14-deoxy-ecdysterone and ponasterone (isomers) at 465.3 *m*/*z*, and for 14-deoxy-poststerone at 347.2 *m*/*z*. For the quantitation of the samples, the ion transition *m*/*z* 481.3 → 445.3 has been used for ecdysterone, *m*/*z* 465.3 → 303.2 for 14-deoxy-ecdysterone, *m*/*z* 465 → 447.3 for ponasterone (ISTD), and finally *m*/*z* 347.2 → 329.1 for 14-deoxy-poststerone.

### 2.1. Validation of the Analytical Methodology

Prior to its application, complete validation of the analytical methodology was performed according to the European Medicines Agency (EMA) [36] and the International Council for Harmonization (ICH M10) [37] guidelines for Bioanalytical Method Validation.

The analytical procedure was validated in terms of selectivity, linearity, limit of detection (LOD), and limit of quantitation (LOQ), precision and accuracy, matrix effect, stability, and carry over.

#### 2.1.1. Selectivity

The selectivity was studied by analyzing six individual blank urine samples to determine if anything in the matrix interfered with the analyte(s) of interest and the internal standard (ISTD). No interfering signals at the retention time of ecdysterone (RT = 2.99), 14α-deoxy ecdysterone (14-deoxy-ecdysterone) (RT = 3.52), 14-deoxy-poststerone (RT = 4.07), and ponasterone (internal standard, ISTD) (RT = 4.81) were detected. Additionally, no interferences with other isomeric ecdysteroids, i.e., ecdysone and 14β-deoxy-ecdysterone, occurred due to their chromatographic resolution from the target analytes.

#### 2.1.2. Linearity of the Calibration Curves, LOD, and LOQ

For the response function, blank urine samples were spiked with ecdysterone at 12 calibration levels from 1 to 5000 ng/mL. In the case of 14-deoxy-ecdysterone and 14-deoxy-poststerone, 10 calibration levels, 1 to 1000 ng/mL, were used. Each level of calibrants was prepared in duplicates. Calibration curves were constructed based on the peak area ratios of the analytes to the ISTD (y-axis) versus the nominal standard concentration (x-axis).

Back calculation was performed to determine the concentrations of ecdysterone, 14-deoxy-ecdysterone, and 14-deoxy-poststerone in each calibration standard, which were used for the quantitation of the analytes in quality control samples (QCs) and post-administration urine samples by applying the equation y = ax^2^ + bx + c, using the Mass Hunter Quant Software Version 10 from Agilent. The weighted quadratic regression was applied after the evaluation of linearity, according to Mandel’s fitting test (F-test), which resulted in a significantly better fit of the second-order calibration function (quadratic), in comparison to the first-order regression function (linear), with *p* = 99%. Furthermore, by testing the homogeneity of variance according to DIN 38402 T51, a significant difference between variances (*p* = 99%) was reported. Consequently, the weighted factor 1/x was applied. The best fit for ecdysterone and 14-deoxy-ecdysterone was indicated by the correlation coefficients (R^2^) of 0.997 and 0.998, respectively.

The LOD was calculated using the standard deviation (SD) of the response and the slope of calibration (LOD = 3.3 × S.D./slope) [38], and corresponded to 0.24 ng/mL for ecdysterone and to 0.34 ng/mL for 14-deoxy-ecdysterone.

The LOQ was determined based on the lowest concentration in which the % error of accuracy was within ±–20% and coefficient of variation (CV) ≤ 20% [37], and corresponded to 1 ng/mL for both ecdysterone and 14-deoxyecdysterone.

Results are reported in Table 1.

#### 2.1.3. Accuracy and Precision

For the evaluation of intra-day accuracy, expressed as the percentage of relative error (RE%) and precision, reported as the percentage of coefficient of variation (CV%), five replicates of the quality control samples (QCs) at the low concentration (LQC), at two different medium concentrations (MQC1 and MQC2), and at high concentration (HQC), were analyzed in the same day. The intermediate precision was evaluated by injecting the LQC, MQC, and HQC in three different days. The results of accuracy, intraday, and intermediate precision for ecdysterone and 14-deoxy-ecdysterone were all within the acceptance values (CV% < 15%, RE < 15%), indicating that the analyte and the metabolite concentration in the urine samples could be determined with reasonable precision and accuracy. Details are reported in Table 2.

#### 2.1.4. Matrix Effect

For the evaluation of the matrix effect, blank urine samples of six different volunteers from individual donors (3 female and male) were analyzed.

The matrix effect was evaluated by spiking ecdysterone and 14-deoxy-ecdysterone at LQC and HQC, in matrix and non-matrix samples. For each analyte and ISTD, the matrix factor (MF) was determined by calculating the ratio of the peak area in the presence of matrix to the peak area, in the absence of matrix (analytes and ISTD spiked in the methanol:water 10:90, *v/v*). The ISTD normalized MF was calculated by dividing the MF of the analytes by the MF of the ISTD. The CV of the ISTD-normalized MF calculated was lower than 15% in all 6 lots of matrix at LQC and HQC, except for 14-deoxy-ecdysterone at the LQC, in which it corresponded to 20%. Details are reported in Table 3. These results showed a high variability. Thus, matrix-matched calibrants were used for quantitation.

#### 2.1.5. Stability

The stability of ecdysterone and 14-deoxy-ecdysterone in urine was determined in triplicates, using the LQC and HQC samples, which were analyzed immediately and again after storage, as reported in Table 4. Freshly prepared QC samples (at t = 0) were used as baseline to assess the stability.

To evaluate the bench-top stability, LQC and HQC samples were left at room temperature for 4, 8, and 24 h, before analysis. Freeze and thaw stability was evaluated after three cycles for LQC and HQC. Long-term stability was evaluated analyzing the LQC and HQC samples, after storing them at −20 °C for two weeks. The results of the stability tests applied were calculated using the peak area ratio of the analytes to the ISTD, which were compared to the baseline (t = 0). Comparing the mean of the ratios after a specific storage condition to the mean of the ratio at t = 0. The results obtained were all within the ±15%. Details are reported in Table 4.

#### 2.1.6. Carry Over

Carry over was tested after the injection of a HQC sample. No signal higher than the 20% of the LOQ for ecdysterone and 14-deoxy-ecdysterone was detected in the blank samples (methanol). Thus, carry-over was considered irrelevant.

### 2.2. Post-Administration Urine Analysis and Evaluation of the Urinary Excretion Profiles of Ecdysterone and Its Metabolites

The elimination of ecdysterone and its metabolites in post-administration urine samples, after a single dose administration of pure ecdysterone to 12 subjects, was evaluated. For calibration, blank urine samples were spiked with increasing concentrations of ecdysterone, 14-deoxy-ecdysterone, and 14-deoxy-poststerone reference standard solutions (matrix-matched standard).

In this study, ecdysterone was detected in all subjects, following the administration of 50 mg of pure ecdysterone. The 14-deoxy-ecdysterone and a new metabolite, the 14-deoxy-poststerone, were detected and confirmed by comparing the retention time and the mass spectra with the reference standard material, using LC–MS/MS.

The developed and validated method enabled the quantitation of ecdysterone and 14-deoxy-ecdysterone in post-administration urine samples and consequently their excretion profiles were evaluated.

Concentration data below the LOQ (but not below the LOD) were included to evaluate the excretion profile, as their inclusion can result in a better fit of the excretion profile model, while excluding or replacing them with zero could lead to biased pharmacokinetic parameters [39,40,41,42]. However, there is an increased uncertainty in the quantitative measurement of these low-level concentrations.

**Figure 2:**
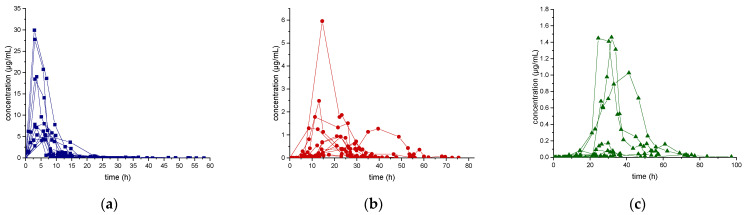
Urinary excretion profile (concentration-time curve) of ecdysterone (**a**), 14-deoxy-ecdysterone (**b**), and 14-deoxy-poststerone (**c**), following a single-dose administration of 50 mg of pure ecdysterone in humans (*n* = 12).

**Figure 3:**
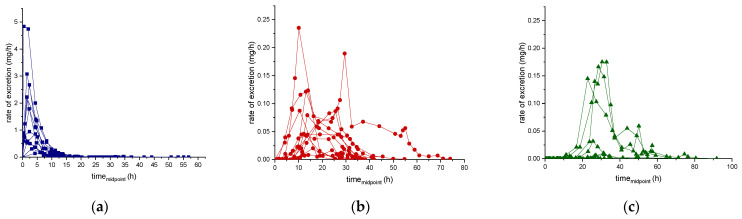
Urinary excretion profile (rate of excretion-midpoint time curve) of ecdysterone (**a**), 14-deoxy-ecdysterone (**b**), and 14-deoxy-poststerone (**c**), following a single-dose administration of 50 mg of pure ecdysterone in humans (*n* = 12).

Results of the excretion profile of ecdysterone, 14-deoxy-ecdysterone, and 14-deoxy-poststerone, considering the concentration versus time and the rate of excretion versus middle-point time curves, are reported in Figure 2 and Figure 3, respectively.

Following a single-dose administration of 50 mg of ecdysterone, the parent compound resulted to be the most abundant analyte in all post-administration urine samples. Its maximum concentration was detected in the 2.8–8.5 h urine, ranging from 4.4–30.0 µg/mL. The maximum excretion rate (mg/h) was detected in the 0.4–4.4 h urine, ranging from 0.1–4.8 mg/h. Ecdysterone was detectable in urine samples, after 45 min from administration and for more than 2 days (58 h).

The metabolite 14-deoxy-ecdysterone was also detectable in all urine samples analyzed. The maximum concentration was detected in the 8.5–39.5 h urine, ranging from 0.1–6.0 µg/mL. The maximum excretion rate was determined in the 10.1–29.5 h urine, ranging from 0.02–0.24 mg/h. 14-Deoxy-ecdysterone was already detected after 2.95 h and remained detectable for about 3 days (75 h).

In contrast to the parent compound and to the 14-deoxy-ecdysterone, 14-deoxy-poststerone was only detected in 10 out of 12 subjects. For 5 volunteers, it was possible to obtain an excretion profile curve.

The maximum concentration was detected in the 23.3–41.3 h urine, ranging from 0.1–1.5 µg/mL. The detection window of 14-deoxy-poststerone ranged from 8.50 to 97 h.

The mean (*n* = 12) of the maximum urinary concentration (C_max_), time to maximum urinary concentration (T_max_), maximum urinary rate of excretion (ER_max_), and maximum urinary middle-point time (midpoint time_max_) were calculated and the details are reported in Table 5.

### 2.3. Evaluation of Urinary Pharmacokinetic Parameters—Cumulative Amount and Half-Life

**Figure 4:**
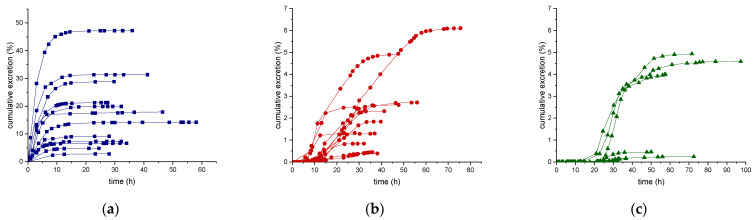
Cumulative urinary excretion curve of ecdysterone (**a**), 14-deoxy-ecdysterone (**b**), and 14-deoxy-poststerone (**c**), following a single-dose administration of 50 mg pure ecdysterone in male and female (*n* = 12).

For each subject considered in this study (*n* = 12), the amount of ecdysterone, 14-deoxy-ecdysterone, and 14-deoxy-poststerone excreted in urine (cumulative amounts, Du), after administration of 50 mg of pure ecdysterone were calculated. The cumulative urinary excretion curves of ecdysterone, 14-deoxy-ecdysterone, and 14-deoxy-poststerone, expressed as percentages relative to the dose administered (50 mg) versus sampling time (hours) are displayed in Figure 4. The cumulative excretion percentages range from 2.8–47.2% for ecdysterone, from 0.4–6.1% for the 14-deoxy-ecdysterone, and from 0.01–4.9% for 14-deoxy-poststerone. These results showed that the cumulative excretion percentages of 14-deoxy-ecdysterone and 14-deoxy-poststerone were much lower than the one obtained for ecdysterone, which was excreted in urine samples, faster than the metabolites (Figure 4).

**Figure 5:**
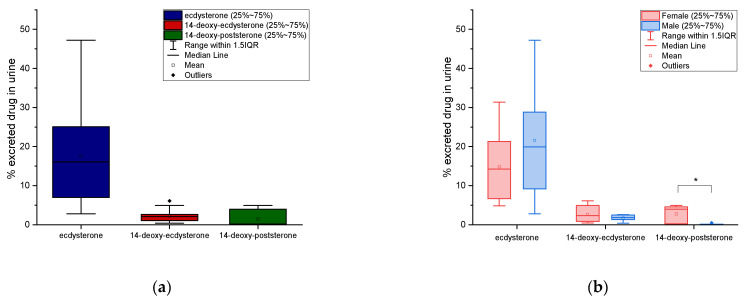
Box-plots of urinary cumulative excretion for ecdysterone, 14-deoxy-ecdysterone, and 14-deoxy-poststerone (**a**) and their comparison in male and female, after administration of 50 mg of ecdysterone (**b**); * significantly different *p* ≤ 0.05 (**b**).

The distributions of cumulative urinary excretion percentage of ecdysterone, 14-deoxy-ecdysterone, and 14-deoxy-poststerone are reported as box-plots in Figure 5. The mean values correspond to 18% with an SD of ±13%, 2.3% ± 1.74%, and 1.5% ± 2.1%, respectively.

A comparison of the urinary cumulative percentages of ecdysterone, 14-deoxy-ecdysterone, and 14-deoxy-poststerone after administration of 50 mg of pure ecdysterone in male and female, was performed using a T-test. No significant differences between male and female (*p* ≤ 0.05) were observed for ecdysterone and 14-deoxy-ecdysterone, while significant differences (*p* ≤ 0.05) were observed for the metabolite 14-deoxy-poststerone. Results are reported in the boxplots in Figure 5.

The half-lifes of ecdysterone, 14-deoxy-ecdysterone, and 14-deoxy-poststerone in post-administration urine samples were calculated using two different methods—rate of excretion method and the sigma-minus method. The calculated half-life for ecdysterone and 14-deoxy-ecdysterone were found to be similar and corresponded to about 3 h. The half-life of 14-deoxy-poststerone instead was much longer using the rate of excretion than the sigma minus method.

**Figure 6:**
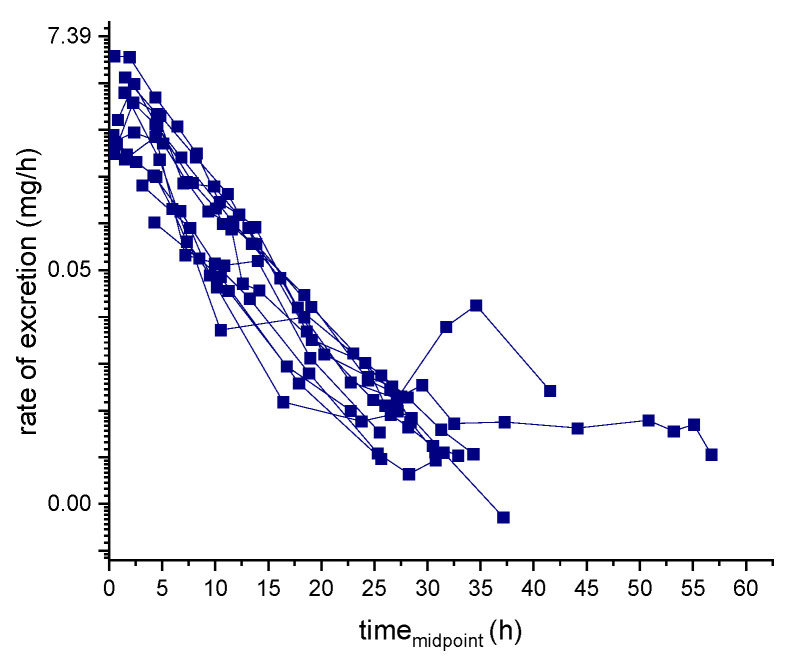
Ecdysterone ln-transformed excretion rate.

Details of the cumulative amount of the analyte and the metabolites excreted in urine samples, as well as the calculated half-life, are reported in Table 6. The ln-transformed excretion rate used to calculate the elimination rate constant (k) of ecdysterone and the corresponding half-life is displayed in Figure 6.

## 3. Discussion

In this study, an LC–MS/MS method was successfully developed for the identification and quantitation of ecdysterone and its metabolites. The analytical method was validated for ecdysterone and 14-deoxy-ecdysterone only. After the administration of a single dose of 50 mg of pure ecdysterone to twelve healthy subjects (five males and seven females), the excretion profile and urinary pharmacokinetic parameters of ecdysterone and its metabolites were evaluated. Ecdysterone was the most abundant analyte detected in post-administration urine samples. Its early detectability in urine (45 min post administration), indicates its rapid absorption and excretion. The kinetics of elimination of ecdysterone was evaluated using the log-linear rate of excretion versus the middle-point time, and corresponded to a first-order.

Ecdysterone was detected in post-administration urine samples for more than 2 days, and its C_max_ in urine was reached in the 2.8–8.5 h. From the results of the cumulative urinary excretion, we can assume that after 12 h, ecdysterone reaches a plateau, indicating that it is almost completely eliminated, the maximum amount of ecdysterone in urine samples is reached, and that it corresponds to 18%. Ecdysterone was found to have a short urinary half-life, which corresponds to 3.4 and 3.0 h, when using the rate of excretion and the sigma-minus method, respectively.

In this study, the presence of the metabolite 14-deoxy-ecdysterone was observed in the post-administration urine samples and its formation was confirmed for all 12 subjects considered. The urinary detection of 14-deoxy-ecdysterone after administration of ecdysterone was previously reported by Brant and Parr et al. in humans [33,34], Kumpun in mice [10], and Destrez in calves [43,44]. Instead, Tsitsimpikou et al. reported the formation of 2-deoxy-ecdysterone and deoxy-ecdysone as urinary metabolites [32]. Kumpun at al. reported the formation of a deoxy-metabolite as caused by gut bacteria [10]. This should be confirmed by further metabolic studies.

The 14-deoxy-ecdysterone was excreted later than the parent compound, probably due to its less polar physico-chemical characteristics. It was detected in post-administration urine samples for about 3 days, reaching the C_max_ in the 8.5–39.50 h urine.

Unlike ecdysterone, that after reaching the maximum excretion rate showed a decline to the base level, in several subjects, a first increase of the excretion rate of 14-deoxy-ecdysterone to a maximum level was followed by a second peak (Figure 3). Thus, it might be assumed that there is a rate-limiting step in the pharmacokinetics process of 14-deoxy-ecdysterone or that it remains longer in other compartments, before it is excreted in urine.

Results from the cumulative excretion of 14-deoxy-ecdysterone, in contrast to ecdysterone, showed that the plateau was reached at different times in the different subjects considered. Specifically, in 58.3% of subjects the plateau was reached between 25–40 h, in 25% it was reached between 15 to 25 h, and in the remaining 16.6% between 40–62 h. These results indicate an inter-individual variability in the formation, absorption, and excretion rate of the metabolite. The maximum amount of 14-deoxy-ecdysterone reached in urine samples corresponded to 2.3% (mean, *n* = 12). As for ecdysterone, a short half-life was also observed for 14-deoxy-ecdysterone, calculated as 3.1 and 2.2 h, using the rate of excretion and the sigma-minus method, respectively.

In humans, 14-deoxy-poststerone was detected as a new metabolite. Its identity was confirmed in post-administration human urine samples, in comparison to authentic reference material. The 14-deoxy-poststerone was detectable in post-administration urine samples for about 4 days and the C_max_ was reached in the 23.3–41.3 h urine. The maximum amount excreted in urine corresponds to 1.5%. In mice, Kumpun et al. [10] already reported it as metabolite of ecdysterone, as well. Unlike the ecdysterone parent compound and 14-deoxy-ecdysterone, 14-deoxy-poststerone was only identified in 10 out of 12 subjects. Analogous to the formation of progestins from cholesterol in human steroid biosynthesis, 14-deoxy-poststerone may be generated by side-chain cleavage. Postulated by Kumpun et al., this may be catalyzed either by a cytochrome P450 enzyme or generated by gut microorganisms [10].

As a consequence, if these reactions are caused by the gut bacteria, individual variability in the metabolic profile needs to be considered. No correlation between the concentration of 14-deoxy-ecdysterone and 14-deoxy-poststerone was observed. To understand whether the latter can be selected as a target metabolite for an ecdysterone administration, further investigations are needed.

The cumulative excretion percentage of 14-deoxy-poststerone reached a plateau between 36 to 61 h post-dosage. Similar to the trend of the excretion rate of 14-deoxy-ecdysterone, a small increase in the excretion rate of 14-deoxy-poststerone, after the maximum peak was achieved, was observed. The half-life of 14-deoxy-poststerone calculated using the excretion rate method corresponded to 9.7 ± 9.5 h, while with the sigma-minus it corresponded to 4.4 ± 1.0 h. This can be explained as a result of the fluctuation observed during the elimination phase, influencing the linearity of the line constructed to obtain the k-value, which is used to calculate the half-life.

The results of the C_max_ obtained in this study showed that there is a high inter-individual variability in the excretion of ecdysterone and its metabolites. Statistical evaluation was conducted to compare the percentage of excreted ecdysterone, 14-deoxy-ecdysterone, and 14-deoxy-poststerone, between females and males. No significant difference was found for ecdysterone and 14-deoxy-ecdysterone, while for 14-deoxy-poststerone, a significant difference (*p* ≤ 0.05) between female and male was observed.

Excretion profile results obtained when using the rate of excretion or the concentration, indicate that the knowledge of urinary information (e.g., volume, collection time) can influence the point in which the highest concentration or excretion rate is detected.

In total, up to 50.3% (mean = 21.1%, *n* = 12) of the administered dose was recovered in urine (as a parent drug and metabolites). The remaining dose might be unabsorbed due to low bioavailability, or might be excreted by other pathways, such as biliary excretion, sweat, saliva, feces, or it can also be transformed to another metabolite that is not yet detected and quantified within this study.

Although the findings of this study provide consistent information regarding the urinary excretion and pharmacokinetics of ecdysterone and its metabolites in human urine, the method was validated only for ecdysterone and 14-deoxy-ecdysterone. For 14-deoxy-poststerone, no full validation was performed. Furthermore, no investigation on the excretion as phase II metabolites was carried out. However, earlier investigations showed no excretion of ecdysterone or 14-deoxy-ecdysterone as conjugates. Further investigations on phase II conjugates of 14-deoxy-poststerone are planned for the future.

## 4. Materials and Methods

### 4.1. Chemicals and Reagents

Reference standards of ecdysterone (2β,3β,14α,20β,22R,25-hexahydroxy-5β-cholest-7-en-6-one, parent compound, **PC**, purity > 95%) was purchased from Steraloids (Newport, RI, USA), while Ponasterone (2β,3β,14α,20β,22R-pentahydroxy-5β-cholest-7-en-6-one, used as internal standard, **ISTD**) was obtained from Santa Cruz Biotechnology, Inc. (Heidelberg, Deutschland). Alpha-14-deoxy-ecdysterone (**M1**) and alpha-14-deoxy-poststerone (**M2**) were purchased from Extrasynthese (Genay CEDEX, France).

### 4.2. Oral Administration of Ecdysterone and Urine Collection

The study was approved by the ethical committee of the German Sport University Cologne, and was carried out following the regulations of the Helsinki declaration. Twelve healthy subjects (7 females and 5 males) with a mean age (standard deviation [SD]) of 26 (3.1) y, weight of 74 (12.0) kg, and height of 174 (8.3) cm participated in the study. A single-dose of 50 mg of pure ecdysterone dissolved in 50 cl of alcoholic solution was administered to the subjects. All doses were administered at 9.00 a.m. in the morning. The urine samples were collected one day before (blank samples) and five days after the administration of ecdysterone. Sampling time (h) and urine volume (mL) were recorded. Aliquots of urine samples were stored frozen at −18 °C, until analysis.

### 4.3. Standard Solutions and Quality Control Samples

Stock solutions of ecdysterone, ponasterone (ISTD), 14-deoxy-ecdysterone, and 14-deoxy-poststerone were prepared in methanol, at a concentration of 1 mg/mL. Working solutions were prepared by diluting the stock solution of ecdysterone, 14-deoxy-ecdysterone, and 14-deoxy-poststerone, in methanol, and were used for preparation of the calibrants. Serial dilution with appropriate amounts of working solutions were prepared in the pooled blank urine samples (matrix-matched standards), to obtain the final concentrations of 1, 2.5, 5, 10, 25, 50, 100, 250, 500, 1000, 2500, and 5000 ng/mL of ecdysterone, and concentrations ranging from 1 to 1000 ng/mL for 14-deoxy-ecdysterone and 14-deoxy-poststerone, to prepare the respective calibration standard ranges, in a final volume of 1 mL. Appropriate amount of a working solution of ponasterone (10 µg/mL) was prepared and used to spike the blank urine samples, obtaining a final concentration of 100 ng/mL in all calibrants.

Quality control (QC) samples were independently prepared at 4 different levels of concentration—for ecdysterone 1 ng/mL (LQC), 250 ng/mL (MQC1), 2500 ng/mL (MQC2), and at 5000 ng/mL (HQC). However, for 14-deoxy-ecdysterone and 14-deoxy-poststerone, the QC samples were prepared at 1 ng/mL (LQC), 50 ng/mL (MQC1), 500 ng/mL (MQC2), and at 1000 ng/mL (HQC).

### 4.4. Sample Preparation

Urine samples (200 µL) spiked with 10 µL of the ISTD ponasterone (working solution 10 µg/mL) and diluted to 1 mL with methanol:water (10:90, *v/v*). The tubes were vortex-mixed and then centrifuged at 9677 RCF for 8 min.

The supernatants were transferred to autosampler vials and analyzed.

### 4.5. Urine Analysis

The analysis of ecdysterone and its metabolite in calibration and urine samples was performed by LC–MS/MS on an Agilent 1200 Infinity series, coupled to an Ultivo triple quadrupole tandem MS system, utilizing a Jet Stream electrospray ionization (ESI) source and an Ion Funnel (Agilent Technologies GmbH, Waldronn, Germany).

#### 4.5.1. Chromatographic Conditions

Chromatographic separation was achieved with an Agilent Eclipse Plus C18 column (2.1 mm × 50 mm, particle size 1.8 µm). The gradient program started at 12% of eluent B and linearly increased to 40% in 4 min, then to 98% in 1.20 min, 0.30 min hold, followed by 0.20 min equilibration at 12% of eluent B. The linear gradient was applied at a flow rate of 0.45 mL/min, resulting in a total run time of 5.7 min plus 1 min for column equilibration. Solvent A comprised of aqueous formic acid (H_2_O:FoOH, 99.9:0.1, *v/v*) and acetonitrile:formic acid (ACN:FoOH, 99.9:0.1, *v/v*) was used as solvent B. The sample injection volume was 5 µL.

#### 4.5.2. Mass Spectrometric Parameters

The mass spectrometer was operated in multiple reaction monitoring (MRM) acquisition mode, using positive ionization (ESI+). The protonated molecular ion [M+H]^+^ for ecdysterone, ponasterone, 14-deoxy-ecdysterone, and 14-deoxy-poststerone, were detected at *m*/*z* 481.3 for ecdysterone, at 465.3 *m*/*z* for ponasterone and 14-deoxy-ecdysterone (isomers), and at 347.2 *m*/*z* for 14-deoxy-poststerone.

Source and MRM optimization were performed using the MassHunter software (Agilent Technologies Inc. Santa Clara, CA, USA). Resulting parameter capillary voltage of 4000 V, nozzle voltage of 500 V, drying gas flow of 5 L/min (nitrogen) at 150 °C, sheath gas flow of 12 L/min (nitrogen) at 375 °C, and nebulizer pressure of 30 psi (nitrogen) were used for the experiments. Table 7 reports the mass spectrometric parameters for the MRM transition of ecdysterone, ponasterone, 14-deoxy-ecdysterone, and 14-deoxy-poststerone. The chromatograms and mass spectra are reported as the supplemental material.

### 4.6. Evaluation of Excretion Profile and Pharmacokinetic Parameters in Urine

In this study, the excretion profile, the cumulative amount excreted in urine, and the half-life of ecdysterone and its metabolites, after a single-dose administration of 50 mg of pure ecdysterone were evaluated according to [45].

To obtain the excretion rates, Equation (1) was applied:

(1) $$E_{\mathrm{rate},i}=\frac{C_{i}\;x\;V_{i}}{\;\left(t_{i}-t_{i-1}\right)}$$

For each urine sample, the calculated concentration (*C*_i_, ng/mL) of ecdysterone, 14-deoxy-ecdysterone, and 14-deoxy-poststerone was adjusted as a function of the dilution factor applied (1:5) and of the volume of urine collected (*V*_i_, mL), obtaining the corresponding amount of drug in urine (expressed in ng), which was divided by the interval between the time values of the sampling and the previous sampling (*t*_i_ − *t*_i−1_).

The excretion profile curves of ecdysterone and its metabolites for each subject were then obtained by plotting the calculated rate of excretion values (ng/h) versus time (middle-point of sample collection, hours) or simply the calculated concentrations (ng/mL) versus time (sampling time, hours). The first collected interval started at 0 h after the oral administration of ecdysterone at 9 a.m.

For each sample, the amount of drug excreted in urine was calculated and added to the amount of drug recovered in the previous urine sample to obtain the cumulative urinary drug excretion, which was plotted in a graph versus the sampling time (hours).

The half-life using the elimination rate constant (*k*) was calculated by applying Equation (2):

(2) $$t_{1/2}=\frac{ln\left(2\right)}{k}$$

Two different methods were used to calculate the elimination rate constant (*k*). In the rate of excretion method, *k* was obtained from the slope of the elimination phase of the ln-transformed excretion curve (Figure 6).

In the sigma minus method, *k* was obtained from the slope of the elimination phase of the ln-transformed remaining drug to be excreted.

Mass Hunter Quant Software from Agilent was used for data acquisition and analysis. Origin Pro 9.1 software (OriginLab Corporation, Northampton, MA, USA) was used for data visualization and statistical treatment of data.

## 5. Conclusions

The elimination of ecdysterone and its metabolites in human urine, following a single-dose administration of 50 mg of pure ecdysterone was evaluated in twelve volunteers, using a validated LC–MS/MS method, by diluting and injecting the urine samples. Ecdysterone was found to be the most abundant compound, excreted in detectable amounts already after 45 min, indicating its rapid absorption and excretion. Ecdysterone was detected in urine samples for more than 2 days, with a half-life of about 3 h. Two metabolites were also detected in urine samples, 14-deoxy-ecdysterone and 14-deoxy-poststerone. These resulted in a later excretion than the parent compound and could been detected in urine samples for about 3 and 4 days, respectively. Thus, the metabolites could be selected as the target analytes for a longer detection time of an ecdysterone intake. The results obtained within this study showed that about 50% of the administered dose was recovered in urine (sum of ecdysterone and the two metabolites).

## Figures and Tables

**Table 1:** Calibration model LOD and LOQ.

Analyte	Calibration Model	Weighted	Calibration Range (ng/mL)	R^2^	LOD (ng/mL)	LOQ (ng/mL)
Ecdysterone	Quadratic	1/x	1–5000	0.997	0.24	1
14-deoxy- ecdysterone	Quadratic	1/x	1–1000	0.998	0.34	1

**Table 2:** Intraday accuracy, intraday precision and intermediate precision of ecdysterone and 14-deoxy-ecdysterone *.

Compound	QC	Concentration (ng/mL)	Intraday (*n* = 15)			Intermediate Precision (*n* = 15)
			Mean Concentration (ng/mL) ± SD	RE (%)	CV (%)	CV (%)
Ecdysterone	LQC	1	0.92 ± 0.11	−7.6	12.2	9.9
	1° MQC	250	248 ± 9.9	−0.7	4.0	2.7
	2° MQC	2500	2470 ± 70	−1.4	2.8	3.9
	HQC	5000	4770 ± 160	−4.7	3.4	3.3
14-deoxy- ecdysterone	LQC	1	1.0 ± 0.1	−3.4	12.3	11.8
	1° MQC	50	49.7 ± 1.2	−0.7	2.4	3.3
	2° MQC	500	507 ± 14	1.4	2.7	3.8
	HQC	1000	949 ± 32	−5.1	3.4	4.5

* Each value is presented as mean ± SD. CV, coefficient of variation; RE, relative error.

**Table 3:** Evaluation of matrix effects of ecdysterone and 14-deoxy-ecdysterone in human urine samples.

Compound	Level	Concentration (ng/mL)	Matrix Effect % (Mean ± SD) *	CV%
Ecdysterone	LQC	1	85 ± 7	8.0
	HQC	5000	94 ± 4	4.7
14-deoxy-ecdysterone	LQC	1	79 ± 16	20
	HQC	1000	90 ± 7	7.5

* Each value is presented as mean ± SD. CV, coefficient of variation.

**Table 4:** Evaluation of the stability of ecdysterone and 14-deoxy-ecdysterone.

		Ecdysterone		14-deoxy-ecdysterone	
		LQC *	HQC *	LQC *	HQC *
**Bench-top**	**Time/Cycle**	**Stability%**	**Stability%**	**Stability%**	**Stability%**
	0 h	100	100	100	100
	4 h	100	101	103	101
	8 h	104	98	101	100
	24 h	100	100	97	101
**Long term**	0	100	100	100	100
	2 weeks	94	87	91	87
**Freeze-Thaw**	0	100	100	100	100
	3 cycles	111	103	110	103

* Each value is presented as mean (*n* = 3).

**Table 5:** Urinary excretion profile parameters calculated as the mean of 12 subject ± standard deviation (SD), after administration of a single dose of 50 mg of pure ecdysterone.

Compound	Excretion Profile Parameters			
	C_max_ (µg/mL)	T_max_ (h)	ER_max_ (mg/h)	Midpoint Time_max_ (h)
Ecdysterone mean ± SD	12.1 ± 9.2	4.6 ± 1.8	1.7 ± 1.4	2.1 ± 1.3
14-deoxy-ecdysterone mean ± SD	1.4 ± 1.6	19.7 ± 8.9	0.1 ± 0.1	17.5 ± 7.0
14-deoxy-poststerone mean ± SD	0.8 ± 0.7 *	30.1 ± 7.2 *	0.1 ± 0.1 *	27.8 ± 3.7 *

* Calculated from *n* = 5.

**Table 6:** Urinary pharmacokinetic parameters calculated as the mean of 12 subject ± standard deviation (SD), after administration of a single dose of 50 mg of pure ecdysterone.

Compound	Cumulative Du (mg)	Half Life (h)	
		Rate of Excretion	Sigma-Minus
Ecdysterone mean ± SD	8.8 ± 6.6	3.4 ± 1.0	3.0 ± 1.0
14-deoxy-ecdysterone mean ± SD	1.1 ± 0.9	3.1 ± 1.3	2.2 ± 0.9
14-deoxy-poststerone mean ± SD	0.7 ± 1.1 **	9.7 ± 9.5 *	4.4 ± 1.0 *

* Calculated from *n* = 5; ** calculated from *n* = 10.

**Table 7:** Mass spectrometric parameters for MRM transitions for ecdysterone, its metabolites, and the ISTD.

Analytes	Retention Time (min)	Precursor Ion (*m*/*z*)	Product Ion (*m*/*z*)	Collision Energy	Polarity
**Ecdysterone**					
quantifier	2.99	481.3	445.3	13	positive
qualifier		481.3	427.3	13	positive
qualifier		481.3	371.2	9	positive
qualifier		481.3	80.9	57	positive
**14-deoxy-ecdysterone**					
quantifier	3.52	465.3	303.2	21	positive
qualifier		465.3	80.9	53	positive
qualifier		465.3	285.2	25	positive
qualifier		465.3	267.2	29	positive
qualifier		465.3	104.9	73	positive
qualifier		465.3	90.9	89	positive
**14-deoxy-poststerone**					
quantifier	4.07	347.2	329.1	16	positive
qualifier		347.2	173.0	28	positive
qualifier		347.2	90.9	68	positive
qualifier		347.2	105.0	56	positive
**Ponasterone**					
quantifier	4.81	465.3	447.3	9	positive
qualifier		465.3	90.9	89	positive
qualifier		465.3	80.9	37	positive

## Data Availability

Raw data are stored by the authors.

## Supplementary Materials

MRM chromatograms and product ions of ecdysterone (a), 14-deoxy ecdysterone (b), 14-deoxy poststerone (c) and ponasterone (d) is available online at https://www.mdpi.com/article/10.3390/metabo11060366/s1.
