# Supplementary figures and images for: Urinary Elimination of Ecdysterone and Its Metabolites Following a Single-Dose Administration in Humans
Source: Metabolites. 2021 Jun 9;11(6):366. doi: 10.3390/metabo11060366 (PMC8227119; doi:10.3390/metabo11060366)

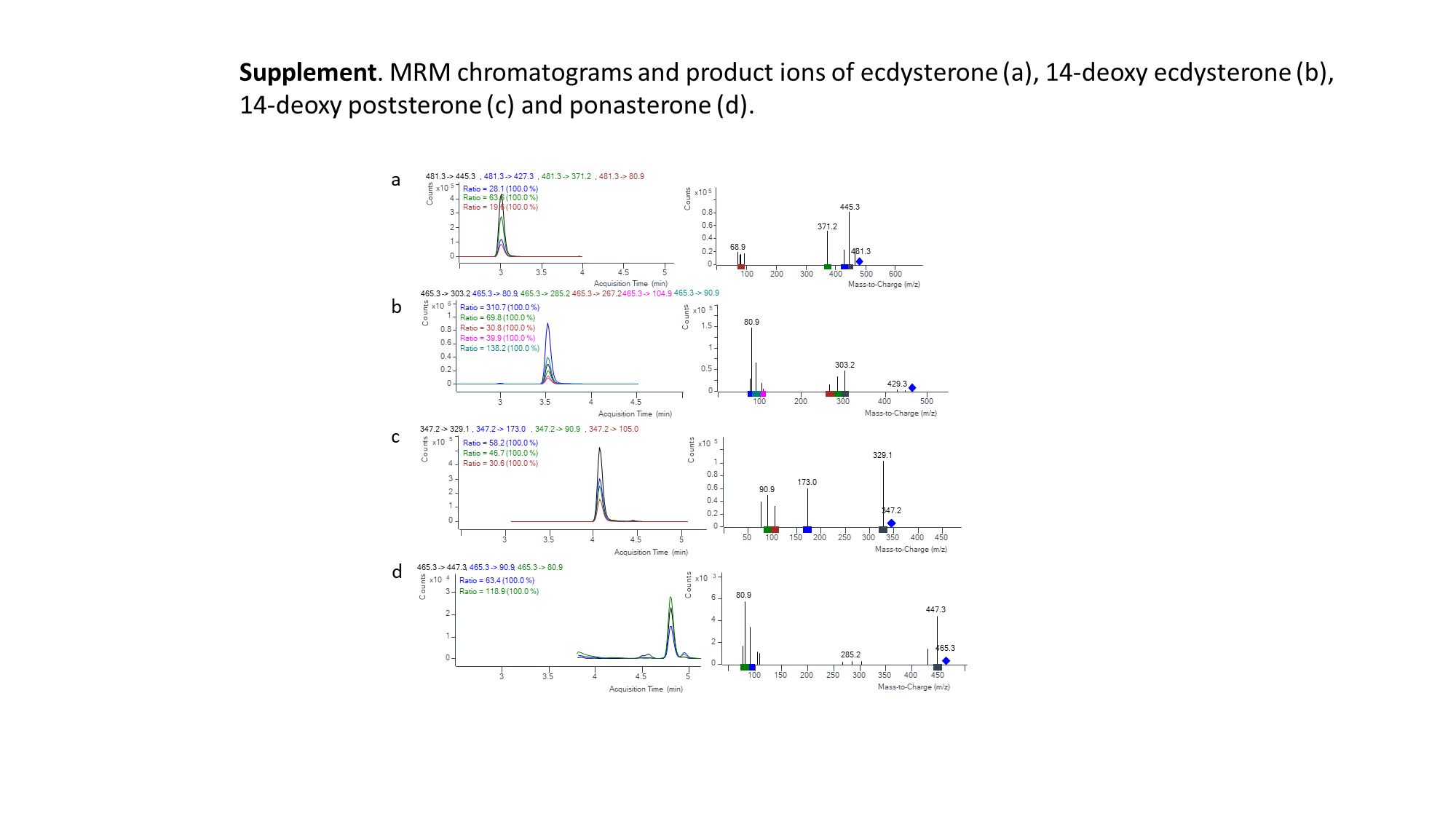

Supplement: Supplementary file 1 [file metabolites-11-00366-s001.zip › metabolites-1224966-SI.TIF]
